# Investigating the Dimensionality of Early Numeracy Using the Bifactor Exploratory Structural Equation Modeling Framework

**DOI:** 10.3389/fpsyg.2021.680124

**Published:** 2021-06-22

**Authors:** Christophe Dierendonck, Anne-Françoise de Chambrier, Annick Fagnant, Christophe Luxembourger, Mélanie Tinnes-Vigne, Débora Poncelet

**Affiliations:** ^1^Department of Education and Social Work, University of Luxembourg, Esch-Belval, Luxembourg; ^2^University of Teacher Education, Vaud, Switzerland; ^3^EQUALE Research Unit, University of Liège, Liège, Belgium; ^4^Laboratoire 2LPN, University of Lorraine, Nancy, France

**Keywords:** early numeracy, early number skills, confirmatory factor analysis, exploratory structural equation modeling, kindergarten, bifactor model, ESEM, structure

## Abstract

The few studies that have analyzed the factorial structure of early number skills have mainly used confirmatory factor analysis (CFA) and have yielded inconsistent results, since early numeracy is considered to be unidimensional, multidimensional or even underpinned by a general factor. Recently, the bifactor exploratory structural equation modeling (bifactor-ESEM)—which has been proposed as a way to overcome the shortcomings of both the CFA and the exploratory structural equation modeling (ESEM)—proved to be valuable to account for the multidimensionality and the hierarchical nature of several psychological constructs. The present study is the first to investigate the dimensionality of early number skills measurement through the application of the bifactor-ESEM framework. Using data from 644 prekindergarten and kindergarten children (4 to 6 years old), several competing models were contrasted: the one-factor CFA model; the independent cluster model (ICM-CFA); the exploratory structural equation modeling (ESEM); and their bifactor counterpart (bifactor-CFA and bifactor-ESEM, respectively). Results indicated acceptable fit indexes for the one-factor CFA and the ICM-CFA models and excellent fit for the others. Among these, the bifactor-ESEM with one general factor and three specific factors (Counting, Relations, Arithmetic) not only showed the best model fit, but also the best coherent factor loadings structure and full measurement invariance across gender. The bifactor-ESEM appears relevant to help disentangle and account for general and specific factors of early numerical ability. While early numerical ability appears to be mainly underpinned by a general factor whose exact nature still has to be determined, this study highlights that specific latent dimensions with substantive value also exist. Identifying these specific facets is important in order to increase quality of early numerical ability measurement, predictive validity, and for practical implications.

## Introduction

### Early Numeracy and the Importance of Its Dimensionality

Early number skills refer to a set of elementary competencies comprising counting, number relations and basic arithmetic operations. These skills have repeatedly been shown to predict more complex mathematical skills as well as general school achievement (Clements and Sarama, 2007, 2011; Duncan et al., 2007; Jordan et al., 2009, 2010). For example, Watts et al. (2014) found that preschool mathematics ability was a significant predictor of math skills up to the age of 15 after accounting for cognitive skills and family background characteristics. It has even been highlighted that early number skills are more strongly related to later math achievement than are early literacy skills to later reading scores, and that early number skills are the strongest predictor of later general school success (Duncan et al., 2007).

Given their importance, early number skills have become the focus of growing interest during these past two decades. Many experts and national commissions have emphasized the necessity of focusing upon early mathematics education (Starkey et al., 2004; National Council of Teachers of Mathematics, 2006; Clements and Sarama, 2007; Ginsburg et al., 2008; National Mathematics Advisory Panel, 2008; National Research Council, 2009; Frye et al., 2013). Indeed, enhancing the early number skills of pupils at (pre)kindergarten can assist children entering with very varied number knowledge, and can help with the process of placing at-risk children onto an appropriate learning trajectory (Ginsburg et al., 2008; Jordan et al., 2010; Scalise et al., 2017). However, despite the growing attention they have received recently, early number skills continue to be studied far less than early reading skills (Mazzocco and Claessens, 2020).

In this context, one major question is to precisely understand the dimensionality of early number skills. Knowing how early numeracy is structured not only allows researchers to assess them more properly, but also to develop more appropriate early intervention strategies. In addition, an accurate identification of the early number skills structure is important to study their relationships with other variables. For example, Brunner (2008) showed that the understanding of the relationships between cognitive abilities and students' characteristics depends strongly on the structural conception of applied cognitive abilities. More precisely, he compared two models of mathematical ability and showed that the correlation between mathematical ability and students' socioeconomic status (SES) was considerably different depending on the model considered. He concluded that in terms of implications for policy makers, results might lead to a decision to invest in specific math interventions with low SES children or might motivate interventions targeting a general factor as well as more domain-specific dimensions (i.e., programs seeking to foster reasoning across education domains, specific mathematical abilities). Thus, without a comprehensive understanding of the early numeracy dimensionality, researchers continue to base their work on unvalidated conceptual frameworks, and risk using a misspecified measurement model which might lead researchers to misleading results and conclusions (Brunner, 2008; Dierendonck et al., 2019; Cimino et al., 2020; Zhang et al., 2020).

In the domain of early literacy, the identification of the set of competencies that support reading acquisition has yielded more effective literacy instruction (Juel and Minden-Cupp, 2000; National Early Literacy Panel, 2008). Regarding numeracy, it is still not clear to date whether specific early numeracy sub-skills exist or are only different means of assessing a general early numeracy construct (Purpura and Lonigan, 2013; Milburn et al., 2019). Consequently, a central question is whether early numeracy skills should be fostered and assessed in a broad perspective, as an overall competence assuming unidimensionality, or whether specific aspects of early numeracy should be considered, assuming multidimensionality.

### Current Knowledge About Early Number Skills Structure

**Figure 1:**
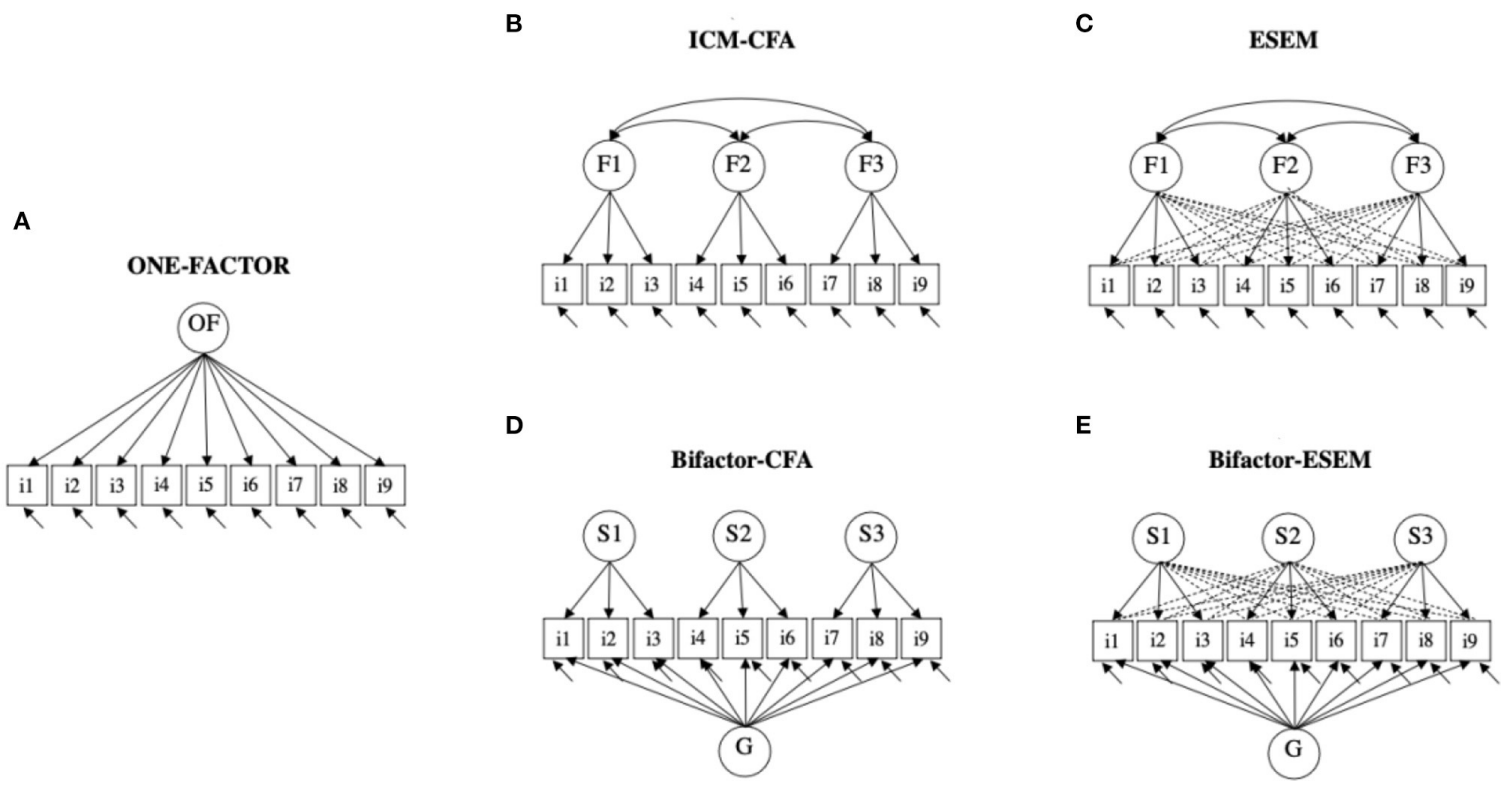
Simplified graphical representations of five alternative models of early numeracy: **(A)** the one-factor model (OF), **(B)** the independent cluster model (ICM-CFA), **(C)** the exploratory structural equation model (ESEM), **(D)** the bifactor-CFA model (Bifactor-CFA), and **(E)** the bifactor exploratory structural equation model (Bifactor-ESEM).

To date, the structure of early number skills has not been the focus of much attention. Interestingly, two of the most important official documents—reports by the National Research Council (2009) and the National Council of Teachers of Mathematics (2006)—do not assume the same number of numeracy sub-dimensions (“Number,” “Relations,” and “Operations” for the NRC and “Numbers” and “Operations” for the NCTM), suggesting a tripartite and a bipartite model of early numeracy, respectively. The few empirical studies that have analyzed the dimensionality of early number skills (see Appendix 1 for a detailed overview) have mainly investigated whether an a priori model was confirmed through confirmatory factor analysis (CFA), in particular, through the one-factor model (OF) for unidimensionality or the independent cluster model (ICM) for multidimensionality. As depicted in Figure 1, the one-factor model (OF) considers only one general ability or dimension. Individual differences are supposed to be due to individual differences on a single common latent factor. The first-order factor model (ICM-CFA) assumes that the measured ability is composed of several first-order specific facets F1, F2 and F3 which may be correlated, but are depicted by independent factors. Although CFA is the most commonly used approach to model construct-relevant multidimensionality, it is often criticized for its very restrictive independent cluster model assumption, which requires that each item to be defined by only one content domain. Under this overly restrictive assumption, factor correlations tend to be inflated (Asparouhov and Muthén, 2009; Morin et al., 2020).

The dimensionality studies of early number skills yielded inconsistent results. In studies that did not consider the hypothesis of a general factor underlying early numeracy, the number of first-order factors varies from 2 to 5. This variation is partly explained by the fact that the researchers used different measurement tools. In the only two studies we are aware of, that consider the hypothesis of a general factor through a bifactor-CFA model (see below), the number of specific factors ranges from 2 (Mou et al., 2021) to 7 (Ryoo et al., 2015).

### How to Capture the Potential Multidimensionality and Hierarchically Nature of Early Numeracy?

#### Bifactor-CFA

In order to take into account the shared communality of each item, the bifactor-CFA (see Figure 1) has been proposed (Reise, 2012). The construct under consideration is assumed to be independently and directly influenced by a general factor and by several specific facets. All factors are supposed to be orthogonal, and the model divides the total observed covariance of the items into a general component (G) which underlies all items, and specific facets (S1, S2, S3…), thus explaining the residual covariance that is not explained by the general factor (Figure 1). The bifactor-CFA better represents the multidimensionality of a construct by taking into account its hierarchical nature, and it is increasingly popular in the literature. As far as we know, only two studies of early number skills have tested a bifactor-CFA representation of their data (see Appendix 1 for details). Mou et al. (2021) focused on set-to-number and number-to-set tasks (Wynn, 1992; Le Corre et al., 2006). They have compared a single-factor model, a 2-factor ICM-CFA and a bifactor-CFA model with a general factor and two specific dimensions. They concluded that set-to-number and number-to-set tasks have substantial overlap but do not appear to be conceptually interchangeable, as the bifactor model showed the best fit to the data. Ryoo et al. (2015) conducted several CFAs (one-factor model, ICM-CFA model, second-order model^1^ and bifactor-CFA model) across four time points and observed inconsistent results. Best fitting models were, respectively, a 7-factor bifactor-CFA for first and second time points, a 7-factor ICM-CFA model for the third time point and an 8-factor ICM-CFA model for the fourth time point. They did not test models with <7 factors. They concluded that when the shared variance between all items is kept under control, several specific factors appear to explain the residual covariance, which suggests that early number skills seem to have a hierarchical structure.

Bifactor-CFA however raises some measurement and theoretical concerns (Morin et al., 2016a; Bonifay et al., 2017; Sellbom and Tellegen, 2019). In particular, the bifactor-CFA neglects the possibility that items may have cross-loadings on the non-target specific factors. Such unmodeled cross-loadings tend to result in inflated G-factor loadings (Murray and Johnson, 2013; Morin et al., 2016a). In order to take the item cross-loadings into consideration, Asparouhov and Muthén (2009) proposed the exploratory structural equation modeling (ESEM) framework as an alternative to CFA models.

#### ESEM and Bifactor-ESEM

ESEM allows for the integration of exploratory factor analysis (EFA) within structural equation modeling (SEM). ESEM with target rotation relies on similarly defined factors as in ICM-CFA, but allows for cross-loadings to be freely estimated between items and non-target factors. Relative to ICM-CFA, ESEM estimates loadings for indicators on all factors, and provides more precision in the estimation of factors and more accurate estimates of factor correlations, which results in better discriminant validity (Asparouhov and Muthén, 2009; Morin et al., 2016a,b). Moreover, when estimated with target rotation, ESEM can be used for purely confirmatory purposes (Asparouhov and Muthén, 2009).

To take into account the advantages of the ESEM framework, as well as the hierarchical nature of some constructs, the bifactor exploratory structural equation modeling (bifactor-ESEM) has recently been proposed by some authors (Jennrich and Bentler, 2011; Morin et al., 2016a,b) (Figure 1). Unlike CFA, this new modeling takes the relations of non-target constructs and items into account, and unlike ESEM, it allows the coexistence of both a latent general construct and specific subdomains. By overcoming the CFA and ESEM shortcomings, the bifactor-ESEM might be the most comprehensive and flexible model, able to describe the complex psychological characteristics with most accuracy (Gu et al., 2020).

Since this pioneering work, the bifactor-ESEM proved to be very interesting in the way it accounts for the dimensionality of several psychological constructs (Fadda et al., 2017; Sánchez-Oliva et al., 2017; Gu et al., 2020). However, neither ESEM nor bifactor-ESEM has already been used to study the dimensionality of early number skills. Now, on the one hand, the structure of this domain should be studied by allowing cross-loadings on non-target specific factors, in order to prevent the G-factor loadings inflation to inflate G-factor loadings (Asparouhov and Muthén, 2009). On the other hand, the high intercorrelations between the first-order factors found in early numeracy (i.e., Purpura and Lonigan, 2013)—which is higher than in literacy for example (Storch and Whitehurst, 2002; Thomas et al., 2018)—might mask a more general factor describing variation in responses across all the observed indicators (Betts et al., 2011; Ryoo et al., 2015). Importantly, while bifactor models tend to show superior goodness of fit in model comparison studies (Bonifay et al., 2017), model comparisons need to be anchored both in theory and in a detailed examination of parameter estimates, as suggested by Morin et al. (2020).

### The Current Study

To our knowledge, no study applied the bifactor-ESEM framework in the field of early numeracy. Given the strong correlations between subdimensions within that domain and the relevance of the bifactor-ESEM framework applied in other areas, the current study aims to investigate the multidimensionality and hierarchical nature of early skills by assuming that a bifactor-ESEM representation is theoretically supported and would better account for numeracy data than rival measurement models. We suggest that our study could serve as a foundation for future studies aiming to examine the dimensionality of early numeracy by using the bifactor-ESEM framework.

## Materials and Methods

### Ethics

The study was reviewed and approved by the Ethics Review Panel of the University of Luxembourg in charge of study coordination. The legal guardian of the pupils provided written informed consent to participate in this study.

### Participants and Procedure

The present article is based on pretest data collected during a quasi-experimental study conducted in four education systems in continental Europe (Belgium, France, Luxembourg and Switzerland), and aimed at measuring the efficacy of a 12 week numerical games intervention with pupils aged from 4 to 6 years (for details and results, see de Chambrier et al., 2021). There were 23 participating schools (46 classrooms) with pupils from mixed socio-economic backgrounds. While 724 children took part in the study, 80 children were not considered, either because they faced major developmental challenges (trisomy/autism), because they were newly arrived in the country and did not speak the school language, or because they were not present on the day of the pretest. The remaining sample of 644 children can be described as follows: mean age of 61.5 months (sd = 7.1) at pretest time, 47.5% girls, 41.6% attending pre-kindergarten, 27.6% from Belgium, 23.8% from France, 28.9% from Luxembourg, and 19.7% from Switzerland.

### Measure

Early numerical ability of children was measured through a test inspired by the TEMA-3 (Ginsburg and Baroody, 2003) and the TEDI-MATH (Van Nieuwenhoven et al., 2001). The TEMA-3 provides a comprehensive assessment of mathematical ability for children aged 3 to 9 years, while the TEDI-MATH enables the diagnosis of mathematical learning disabilities of children aged 5 to 8. These two validated instruments were selected for their psychometrical quality shown in the French-speaking (for the TEDI-MATH) and in the English-speaking (for the TEMA-3) contexts. Relatively to the original tests, the subtests included in the instrument used in the current study were mainly a function of the age of our participants (4 to 6 year old). Overall, the subtests of the French test that were intended to children of our participants' age group (from 5 to 6 years old) were retained and were completed by subtests intended to younger children (from 4 to 5 years old) selected and translated from the English test. Content and descriptive statistics of the 41 items^2^ across the three theoretical sub-categories (counting, number relations, arithmetic) are reported in Table 1. It has to be noted that some items (marked with an asterisk) were only administered if students were successful in previous items.

**Table 1:** Description of the items used to measure early numerical ability.

**Dimensions**	**Items**	**Description**		**% of correct answer**	***N***
Counting	1_1	Verbal counting	Children are asked to count as far as possible (they are stopped at 42)	20.5—Unable to count from 1 to 10 44.2—Able to count from 1 to 10 18.0—Counting error between 11 and 20 9.6—Counting error between 21 and 30 7.6—Counting error after 30	132 284 116 62 49
	1_2	Verbal counting	Count up to 6	70.8	643
	1_3	Verbal counting	Count up to 9	62.3	644
	1_4	Verbal counting	Count from 3	37.6	644
	1_5	Verbal counting	Count from 7	39.0	644
	1_6	Verbal counting	Count from 4 to 8	35.9	644
	1_7	Verbal counting	Count from 7 to 10	34.6	644
	2_1	Counting objects	Children have to enumerate linear collection of 8 aligned rabbits	80.6	644
	2_2	Counting objects	After rabbits have been hidden with a piece of paper, children have to say how many rabbits are hidden (answer is considered as correct if the cardinal is the same as the one given in the previous item, even if this one was wrong)	58.9	644
	2_3	Counting objects	Children have to enumerate dispersed collection of 6 sheep	82.3	644
	2_4^*^	Counting objects	Children have to enumerate dispersed collection of 12 pigs	42.5	577
	3_1	Counting objects	Children have to give 5 tokens to the experimenter	71.1	644
	3_2	Counting objects	Children have to give 8 tokens to the experimenter	55.0	644
	3_3^*^	Counting objects	Children have to give 15 tokens to the experimenter	42.6	472
	4_1	Counting objects	5 farmers are presented each with a hat. After asking the children to close their eyes, the experimenter takes off the hats. Children have to say how many hats are in the hand	68.2	644
	4_2	Counting objects	7 farmers are presented on a piece of paper. Children have to fetch a rake for each farmer. More than seven rakes are located on a separate table	46.1	644
Relations	5_1	Conservation of number	Children are shown one row of 6 tokens that were previously enumerated and are asked how many there are after they are moved into a circle	55.0	644
	5_2	Conservation of number	Children are shown two rows of 7 tokens facing each other and after the experimenter moved items of one row closer together, children have to say whether there is still the same number of tokens or not	27.5	644
	6_1	Seriation	Children are asked to put 3 cards with 6, 1, and 3 frogs from the smallest to the largest quantity	58.9	643
	6_2^*^	Seriation	Children are asked to put 6 cards with 7, 3, 1, 6, and 9 frogs from the smallest to the largest quantity	51.2	379
	6_3^*^	Seriation	Children are asked to put the card with 5 frogs in the right place when cards with 1, 3, 6, and 9 frogs are ordered from the smallest to the largest quantity	50.7	379
	7_1	Numerical inclusion	A closed box with 6 rabbits inside is presented to the children who are asked if there are enough to take 8 of them	53.9	644
	7_2	Numerical inclusion	A closed box with 6 rabbits inside is presented to the children who are asked if there are enough to take 4 of them	68.3	644
	7_3	Numerical inclusion	A closed box with 6 rabbits inside is presented to the children who are asked if there are enough to take 7 of them	51.9	644
	8_1	Magnitude comparisons	Children are shown two collections of elements varying in size and differently dispersed and have to say on which side there are more (8 vs. 7 dots similarly dispersed and of same size)	69.4	644
	8_2	Magnitude comparisons	Children are shown two collections of elements varying in size and differently dispersed and have to say on which side there are more (6 closely spaced vs. 6 distant dots of same size)	34.8	644
	8_3	Magnitude comparisons	Children are shown two collections of elements varying in size and differently dispersed and have to say on which side there are more (7 closely spaced and smaller dots vs. 6 distant and larger dots)	39.6	644
	8_4	Magnitude comparisons	Children are shown two collections of elements varying in size and differently dispersed and have to say on which side there are more (8 distant and smaller dots vs. 8 closely spaced and larger dots)	30.4	644
Arithmetic	9_1	Additions of concrete elements	Experimenter takes a small number of tokens in each hand, shows them to the children, hides both quantities in one hand and asks the children to say how many there are in all (2 and 1)	75.5	644
	9_2	Additions of concrete elements	Experimenter takes a small number of tokens in each hand, shows them to the children, hides both quantities in one hand and asks the children to say how many there are in all (3 and 2)	46.0	644
	9_3	Additions of concrete elements	Experimenter takes a small number of tokens in each hand, shows them to the children, hides both quantities in one hand and asks the children to say how many there are in all (5 and 3)	27.2	644
	9_4^*^	Additions of concrete elements	Experimenter takes a small number of tokens in each hand, shows them to the children, hides both quantities in one hand and asks the children to say how many there are in all (7 and 5)	24.6	175
	10_1	Story problems	There were 4 hens in a courtyard; 3 more arrived; how many are there in total?	36.8	644
	10_2	Story problems	There are six birds on a tree. Two are flying away. How many are left?	26.2	644
	10_3	Story problems	There are 8 sheep in the meadow. Five sheep are white; the others are black. How many black sheep are there?	12.7	644
	11_1	Number decomposition	Experimenter shows a certain number of rabbits to the children, hides some of them while children close their eyes, after which children have to say how many are hidden by looking at the remaining elements (5 are 2 and…)	30.6	644
	11_2	Number decomposition	Experimenter shows a certain number of rabbits to the children, hides some of them while children close their eyes, after which children have to say how many are hidden by looking at the remaining elements (5 are 4 and…)	39.9	644
	11_3	Number decomposition	Experimenter shows a certain number of rabbits to the children, hides some of them while children close their eyes, after which children have to say how many are hidden by looking at the remaining elements (9 are 5 and…)	17.2	644
	11_4	Number decomposition	Experimenter shows a certain number of rabbits to the children, hides some of them while children close their eyes, after which children have to say how many are hidden by looking at the remaining elements (9 are 3 and…)	11.3	644
	11_5^*^	Number decomposition	Experimenter shows a certain number of rabbits to the children, hides some of them while children close their eyes, after which children have to say how many are hidden by looking at the remaining elements (13 are 9 and…)	12.5	369
	11_6^*^	Number decomposition	Experimenter shows a certain number of rabbits to the children, hides some of them while children close their eyes, after which children have to say how many are hidden by looking at the remaining elements (13 are 6 and…)	8.2	368

* *Items that were only administered if students were successful in previous items*.

### Data Analysis

To test our hypothesis, we followed the procedure recommended by Morin et al. (2016a,b). These authors suggest making an initial assessment of the first source of construct-relevant multidimensionality due to the fallible nature of items that include at least some degree of association with non-target dimensions (i.e., cross-loadings). This corresponded to comparing ICM-CFA and ESEM models with one (Aunio et al., 2004), two (National Research Council, 2009; Aunio and Niemivirta, 2010) or three (National Council of Teachers of Mathematics, 2006; Purpura and Lonigan, 2013; Aunio et al., 2019; Milburn et al., 2019) sub-dimensions. ESEM analyses were conducted using a confirmatory approach to rotation (i.e., target rotation), which allows for the a priori specification of indicators of each factor, and for the free estimation of cross-loadings targeted to be as close to zero as possible (Asparouhov and Muthén, 2009). Subsequently, Morin et al. (2016a,b) recommend assessing the second source of construct-relevant multidimensionality due to the potential hierarchical nature of the construct being investigated. This meant comparing the bifactor-counterpart (bifactor-CFA or bifactor-ESEM) of the best fitting first-order solution (for more details, see below). According to this procedure, the bifactor-ESEM solution would have to be retained if the ESEM solution better accounts for the data than the CFA model, and if the bifactor-ESEM solution better fits the data than the first-order ESEM solution.

All analyses were conducted with Mplus 8.4 (Muthen and Muthen, 2012–2019) on the 40 items. Full information robust maximum likelihood (FIML) estimation (Enders, 2010) was used to handle the missing data at item level. As the test included binary coded items except the first one (1_1), the weighted least squares mean and variance adjusted (WLSMV) estimator was used for the factorial analyses. In order to take into account the hierarchical nature of the data (students are nested in classroom), we applied the Mplus design-based adjustment implemented by the TYPE=COMPLEX function (Asparouhov, 2005). For estimating model fit, we followed typical interpretation guidelines (Hu and Bentler, 1999; Marsh et al., 2004) by using several common fit indices: the chi-square statistic (although this statistic is highly sensitive to sample size), the root mean square error of approximation (RMSEA) with its 90% confidence interval, the comparative fit index (CFI), the Tucker-Lewis index (TLI) and the standardized root mean square residual (SRMR). RMSEA must be below 0.06 for an excellent model fit and below 0.08 for an acceptable fit. CFI and TLI must be above 0.95 for an excellent fit and above 0.90 for an acceptable fit. The acceptable range of SRMR is between 0 and 0.08.

Complementary to model fit considerations and as mentioned earlier, Morin et al. (2016a,b) suggested to analyze parameter estimates by starting with a comparison between the ICM-CFA and ESEM solutions. If factors are well-defined by strong target factor loadings in the ESEM solution, the focus has then to be put on the factor correlations matrix. If a discrepant pattern of factor correlations is observed between ICM-CFA and ESEM, the latter model should be retained. Otherwise, parsimony principle argues for retaining the ICM-CFA model. Once the optimal first-order solution has been retained, then Morin et al. (2016a,b) suggested comparing this solution with its bifactor counterpart (respectively, the bifactor-CFA or the bifactor-ESEM). The bifactor representation has to be retained if general and specific dimensions are well-defined and, in the case of an ESEM framework, if cross-loadings decrease in the bifactor-ESEM solution compared to the ESEM solution. For all models, model based composite reliability coefficient calculated from the model standardized parameter estimated as McDonald' (1970) omega (ω) coefficient was reported. According to Perreira et al. (2018), minimal level of acceptability of omega reliability coefficients are 0.60 for measures corresponding to first-order models and 0.50 for measures related to bifactor models.

Measurement invariance across gender (girls/boys) was tested following the procedure described by Toth-Kiraly and Neff (2021) based on Millsap (2011). Nevertheless, as our data includes only binary items, except one, test of the weak invariance level was impossible. More precisely, we successively tested invariance on the configural, strong, strict, latent covariance-variance, and latent means levels. As noted by Brown (2015) and Little (2013), in the context of invariance testing, the chi-square difference test is too sensitive to sample size and to trivial fluctuations and differences. Therefore, alternative fit measures (ΔRMSEA, ΔCFI, and ΔTLI) were used to compare the models. The guidelines suggested by Chen (2007) were followed in comparing nested invariance models (ΔRMSEA ≤ 0.015, ΔCFI ≤ 0.010). TLI change analysis with guidelines similar to CFI was also conducted for purposes of parsimony, as recommended by Marsh et al. (2009).

As the literature does not agree on the number of dimensions, factorial solutions with one, two and three dimensions were compared. With reference to models depicted in Figure 1 and following suggestions made by Morin et al. (2016a,b), eight alternative models were compared: the one-factor model (Model 1), four ICM-CFA models with 2 to 3 factors (Models 2 to 5), the ESEM model (Model 6) related to the best-fitting ICM-CFA model, and the bifactor counterpart of the best-fitting ICM-CFA and ESEM model, respectively, the bifactor-CFA (Model 7) and the bifactor-ESEM (Model 8). Model 2 tested the theoretical two-factor structure with counting/number relations as the first sub-dimension and arithmetic as the second sub-dimension. Model 3 tested the theoretical two-factor structure with counting as the first sub-dimension and number relations/arithmetic as the second sub-dimension. Model 4 tested the theoretical two-factor structure with number relations as the first sub-dimension and counting/arithmetic as the second sub-dimension. Model 5 tested the theoretical three-factor structure with counting as the first sub-dimension, number relations as the second sub-dimension, and arithmetic as the third sub-dimension.

## Results

### Alternative Representations of Early Numeracy

Estimation procedures for all CFA and ESEM solutions converged properly. Fit indices of the rival models of early numeracy are reported in Table 2. Models 1 to 5 showed acceptable but not excellent fit indexes, as CFI and TLI were below 0.95. SRMR for these models was above 0.08. Among ICM-CFA solutions, Model 5 (with 3 correlated factors) was retained due to its superior fit and contrasted with alternative models. Models 6 and 7 showed excellent empirical fit for all indices, except for SRMR (0.081 and 0.083, respectively). The best fit indices were observed for the bifactor-ESEM solution (*Model 8*: CFI = 0.977; TLI = 0.972; RMSEA = 0.022; SRMR = 0.070), demonstrating a higher level of fit to the data than the ESEM Model 6 (ΔCFI = +0.013; ΔTLI = +0.014; ΔRMSEA = −0.005) and the bifactor-CFA Model 7 (ΔCFI = +0.010; ΔTLI = +0.009; ΔRMSEA = −0.004). As model parameter estimates have to be analyzed before any decision, factor correlations for the ICM-CFA Model 5 (|*r*| = 0.778 to 0.899, *M*_|r|_ = 0.856) and the ESEM Model 6 (|*r*| = 0.161 to 0.539, *M*_|r|_ = 0.344) are reported in Table 3. A higher adequacy of the ESEM representation is suggested since factor correlations were considerably reduced between CFA and ESEM solutions. Standardized parameter estimates for Model 5 to 8 are reported in Table 4, but we only compare below the ESEM representation with its bifactor counterpart, as they showed their superior adequacy over CFA models.

**Table 2:** Goodness-of-fit indices for alternative models of early numeracy.

**Models**	**Description**	**Chi-square**	**df**	**CFI**	**TLI**	**RMSEA**	**RMSEA 90% CI**	**SRMR**
1. One-factor model	General	1607.084^**^	740	0.902	0.897	0.043	[0.040,0.046]	0.111
2. ICM-CFA with 2 first-order factors	(C and R); A	1553.403^**^	739	0.908	0.903	0.041	[0.039,0.044]	0.109
3. ICM-CFA with 2 first-order factors	C; (R and A)	1541.217^**^	739	0.910	0.905	0.041	[0.038,0.044]	0.109
4. ICM-CFA with 2 first-order factors	(C and A); R	1602.091^**^	739	0.903	0.897	0.043	[0.040,0.046]	0.111
5. ICM-CFA with 3 first-order factors	C; R; A	1521.940^**^	737	0.912	0.906	0.041	[0.038,0.044]	0.108
6. ESEM with 3 first-order factors	C; R; A	982.139^**^	663	0.964	0.958	0.027	[0.024,0.031]	0.081
7. Bifactor-CFA with 1 general factor and 3 specific factors	General; Specific C; Specific R; Specific A	996.089^**^	700	0.967	0.963	0.026	[0.022,0.029]	0.083
8. Bifactor-ESEM with 1 general factor and 3 specific factors	General; Specific C; Specific R; Specific A	826.022^**^	626	0.977	0.972	0.022	[0.018,0.026]	0.070

** *p ≤ 0.001*.

*p ≤ 0.01. C, Counting; R, Relations; A, Arithmetic; CFA, Confirmatory Factor Analysis; ESEM, Exploratory Structural Equation Model; df, degrees of freedom; CFI, Comparative Fit Index; TLI, Tucker-Lewis Index; RMSEA, Root Mean Square Error of Approximation; CI, Confidence Interval; SRMR, Standardized Root Mean square Residual.

**Table 3:** Standardized factor correlations for the ICM-CFA solutions with 3 first-order factors (above the diagonal) and the corresponding ESEM solutions (below the diagonal).

	**Model 5**		
	**C**	**R**	**A**
**Model 6**	**C**		0.899^**^	0.778^**^
	**R**	0.539^**^		0.892^**^
	**A**	0.333^**^	0.161^**^	

*p ≤ 0.05;

** *p ≤ 0.01. CFA, Confirmatory Factor Analysis; ESEM, Exploratory Structural Equation Model; C, Counting; R, Relations; A, Arithmetic*.

**Table 4:** Standardized factor loadings (λ), uniquenesses (δ) and omega (ω) for Model 5 to Model 8.

**Dimensions/** **Items**	**ICM-CFA** **Model 5**		**ESEM** **Model 6**				**Bifactor-CFA** **Model 7**			**Bifactor-ESEM** **Model 8**				
	**λ**	**δ**	**C (λ)**	**R (λ)**	**A (λ)**	**δ**	**G (λ)**	**S (λ)**	**δ**	**G (λ)**	**S-C (λ)**	**S-R (λ)**	**S-A (λ)**	**δ**
**Counting**														
1_1	0.814	0.337	0.746^**^	0.144^**^	−0.056	0.334	0.712^**^	0.391^**^	0.340	0.712^**^	0.392^**^	0.069	0.105^**^	0.569
1_2	0.925	0.144	0.915^**^	0.058	−0.060	0.136	0.818^**^	0.423^**^	0.152	0.771^**^	0.500^**^	0.176	−0.043	0.350
1_3	0.903	0.185	0.911^**^	0.059	−0.096^**^	0.158	0.794^**^	0.431^**^	0.184	0.753^**^	0.506^**^	0.149	0.023	0.393
1_4	0.860	0.260	0.862^**^	−0.066	0.100^*^	0.248	0.662^**^	0.596^**^	0.207	0.703^**^	0.457^**^	−0.270^**^	0.118	0.459
1_5	0.832	0.307	0.855^**^	−0.077	0.076	0.287	0.609^**^	0.655^**^	0.200	0.654^**^	0.480^**^	−0.327^**^	0.195^*^	0.444
1_6	0.929	0.136	0.845^**^	−0.022	0.223^**^	0.132	0.781^**^	0.509^**^	0.131	0.803^**^	0.406^**^	−0.287^**^	0.017	0.329
1_7	0.904	0.183	0.823^**^	−0.017	0.212^**^	0.178	0.744^**^	0.526^**^	0.170	0.778^**^	0.383^*^	−0.341^**^	0.073	0.355
2_1	0.783	0.387	0.888^**^	−0.010	−0.214^**^	0.301	0.622^**^	0.541^**^	0.320	0.609^**^	0.538^**^	0.231^**^	0.072	0.529
2_2	0.729	0.468	0.619^**^	0.110^*^	0.091	0.483	0.684^**^	0.221^**^	0.484	0.658^**^	0.301^**^	0.084	−0.080	0.681
2_3	0.799	0.362	0.876^**^	0.019	−0.195^**^	0.291	0.652^**^	0.506^**^	0.319	0.639^**^	0.511^**^	0.254^**^	0.054	0.513
2_4	0.487	0.763	0.356^**^	0.228^**^	−0.050	0.747	0.454^**^	0.150^**^	0.771	0.461^**^	0.142^**^	0.014	0.177^**^	0.858
3_1	0.878	0.229	0.828^**^	0.081	0.019	0.225	0.816^**^	0.311^**^	0.238	0.770^**^	0.415^**^	0.175	−0.103	0.441
3_2	0.841	0.292	0.789^**^	0.098	−0.016	0.293	0.758^**^	0.347^**^	0.304	0.730^**^	0.421^**^	0.084	0.004	0.532
3_3	0.550	0.697	0.388^**^	0.279^**^	−0.057	0.672	0.510^**^	0.179^**^	0.708	0.522^**^	**0.137**	−0.056	0.250^**^	0.802
4_1	0.616	0.620	0.439^**^	0.131^*^	0.196^**^	0.624	0.618^**^	0.052	0.616	0.585^**^	0.155^*^	0.003	−0.133^*^	0.784
4_2	0.668	0.554	0.470^**^	0.130^*^	0.238^**^	0.556	0.663^**^	0.072	0.555	0.635^**^	0.170^**^	−0.066	−0.127^*^	0.740
ω	0.964		0.960					0.860			0.799			
**Relations**														
5_1	0.787	0.380	0.549^**^	0.205^**^	0.179^**^	0.427	0.790^**^	0.054	0.373	0.729^**^	0.213^**^	**0.021**	−0.084	0.645
5_2	0.469	0.780	0.080	0.345^**^	0.264^**^	0.731	0.463^**^	0.145^**^	0.764	0.504^**^	−0.117	**−0.112**	0.009	0.848
6_1	0.504	0.746	0.637^**^	−0.332^**^	0.575^**^	0.198	0.713^**^	−0.627^**^	0.099	0.595^**^	0.317^*^	−0.473^**^	−0.389^*^	0.412
6_2	0.413	0.829	0.222^**^	0.697^**^	−0.272^**^	0.325	0.566^**^	0.615^**^	0.302	0.613^**^	0.006	0.594^**^	0.204	0.480
6_3	0.474	0.775	0.143^*^	0.747^**^	−0.206^**^	0.333	0.560^**^	0.611^**^	0.314	0.628^**^	−0.069	0.502^**^	0.261	0.530
7_1	0.692	0.521	0.306^**^	**0.087**	0.595^**^	0.378	0.691^**^	−0.293^**^	0.437	0.644^**^	0.040	**0.125**	−0.522^**^	0.544
7_3	0.654	0.572	0.224^**^	**0.169**	0.571^**^	0.439	0.656^**^	−0.221^**^	0.520	0.627^**^	−0.028	**0.169**	−0.476^**^	0.593
8_1	0.511	0.739	0.525^**^	**−0.127**	0.155^**^	0.708	0.516^**^	−0.214^**^	0.688	0.419^**^	0.294^**^	**0.086**	−0.263^**^	0.813
8_2	0.553	0.694	−0.189^**^	0.778^**^	0.244^**^	0.427	0.490^**^	0.585^**^	0.418	0.614^**^	−0.324^**^	**−0.232**	0.290^**^	0.617
8_3	0.689	0.525	0.364^**^	0.325^**^	0.177^**^	0.542	0.692^**^	0.108^*^	0.509	0.676^**^	0.068	**−0.047**	0.037	0.731
8_4	0.583	0.660	−0.238^**^	0.868^**^	0.327^**^	0.267	0.537^**^	0.628^**^	0.317	0.677^**^	−0.396^**^	**−0.251**	0.293^**^	0.486
ω	0.847			0.821				0.780				0.505		
**Arithmetic**														
9_1	0.748	0.441	0.522^**^	0.114	0.160^*^	0.563	0.693^**^	−0.158^*^	0.495	0.633^**^	0.187^*^	0.161^*^	−0.210^**^	0.703
9_2	0.752	0.435	0.357^**^	0.272^**^	0.279^**^	0.525	0.699^**^	−0.124	0.495	0.687^**^	0.041	−0.005	**−0.127**	0.715
9_3	0.792	0.372	0.283^**^	0.303^**^	0.452^**^	0.403	0.736^**^	−0.343^**^	0.341	0.736^**^	−0.006	−0.016	−0.257^**^	0.626
9_4	0.019	1.00	0.593^**^	0.043	−0.627^**^	0.482	0.316^**^	0.674^**^	0.446	0.207^**^	0.368^**^	0.074	0.567^**^	0.703
10_1	0.818	0.331	0.373^**^	0.379^**^	0.212^**^	0.441	0.760^**^	0.025	0.422	0.752^**^	0.039	−0.020	**0.004**	0.658
10_2	0.512	0.738	0.210^**^	0.235^**^	0.180^**^	0.777	0.473^**^	0.101	0.766	0.479^**^	−0.013	−0.038	**−0.031**	0.876
10_3	0.448	0.800	0.162	0.212^*^	0.190^**^	0.822	0.416^**^	0.105	0.816	0.426^**^	−0.033	−0.043	**−0.046**	0.902
11_1	0.496	0.754	0.030	0.375^**^	0.295^**^	0.718	0.460^**^	0.049	0.786	0.514^**^	−0.190^**^	−0.047	**−0.056**	0.833
11_2	0.622	0.613	0.111^*^	0.467^**^	0.246^**^	0.598	0.580^**^	0.121^*^	0.649	0.631^**^	−0.167^**^	−0.001	**0.015**	0.757
11_3	0.415	0.828	0.143^*^	0.361^**^	**−0.071**	0.804	0.360^**^	0.460^**^	0.659	0.387^**^	−0.050	−0.018	0.293^**^	0.873
11_4	0.515	0.735	0.187^*^	0.497^**^	**−0.154**	0.638	0.460^**^	0.609^**^	0.417	0.495^**^	−0.061	0.077	0.396^**^	0.767
11_5	0.349	0.878	0.253^*^	0.207^*^	−0.181^*^	0.846	0.287^**^	0.519^**^	0.648	0.300^**^	0.044	−0.109	0.382^**^	0.866
11_6	0.440	0.807	0.383^**^	0.365^**^	−0.461^**^	0.529	0.371^**^	0.606^**^	0.495	0.371^**^	0.152^*^	0.234^*^	0.511^**^	0.724
ω	0.730				0.602		0.973	0.671		0.958			0.456	

*C, Counting; R, Relations; A, Arithmetic; G, General dimension; S, Specific facet*.

* *p ≤ 0.05*;

** *p ≤ 0.01. CFA, Confirmatory Factor Analysis; ESEM, Exploratory Structural Equation Model. Target factor loadings are in bold*.

In the bifactor-ESEM solution, the loadings on the general dimension revealed a well-defined G-factor (|λ| =0.207 to 0.803; *M*_|λ|_ = 0.603; ω = 0.958). Loadings on the G-factor were high for items associated with counting/enumeration (|λ| = 0.461 to 0.803; *M*_|λ|_ = 0.674) and lower for items assessing number relations (|λ| = 0.419 to 0.729, *M*_|λ|_ = 0.611) and arithmetic (|λ| = 0.207 to 0.752, *M*_|λ|_ = 0.509). The specific counting dimension was well-defined (|λ| = 0.137 to 0.538; *M*_|λ|_ = 0.370; ω = 0.799) with 15 items on 16 showing a statistically significant loading, but four counting items (1_4 to 1_7) showed negative loading over 0.250 on the specific relations dimension. The specific relations dimension was relatively poorly defined (|λ| = 0.021 to 0.594; *M*_|λ|_ = 0.237; ω = 0.505) with only 3 items on 11 showing a statistically significant loading. Here again, several items targeting the specific relations dimension (6_1, 7_1, 7_3, 8_1, 8_2, 8_4) showed significant cross-loadings over 0.250 on the specific arithmetic dimension. These unexpected significant cross-loadings regarding the specific counting and the specific relations dimensions suggested that some items of the test were maybe measuring other very specific counting and relations facets of early number skills (see the discussion section). The specific arithmetic dimension was not better defined (|λ| = 0.004 to 0.567; *M*_|λ|_ = 0.223; ω = 0.456) with 7 of the 13 target items showing a statistically significant saturation and reasonable omega just below the 0.50 satisfactory minimal level suggested by Perreira et al. (2018). In this case, only one item (9_4) showed a statistically significant loading over 0.250 on a non-target specific dimension. Here, the fact that the factor loading of several target items are not significant on the specific arithmetic dimension and the absence of significant cross-loadings reflects that the variance included in these items were mainly used in defining the G-factor. Comparison between the factor loadings of the ESEM solution and the bifactor-ESEM solution showed that cross loadings tend to be significantly less numerous and smaller in the bifactor-ESEM solution, suggesting the presence of an unmodeled general dimension reflected by the numerous and higher cross loadings in the ESEM solution. More precisely, there were 57 statistically significant cross loadings in the ESEM solution while only 29 statistically significant cross loadings remained in the bifactor-ESEM solution. For all these reasons, we considered that the bifactor-ESEM solution (Model 8) was the best representation of our early numeracy data. The explained common variance (ECV) for this model was 0.75, meaning that the general factor explained 75% of the common variance extracted with 25% of the common variance spread across specific factors.

### Measurement Invariance

To verify the extent to which the bifactor-ESEM representation of early numeracy was the same for boys and girls, we conducted five tests of measurement invariance across gender groups (Models G1 to G5). Goodness-of-fit results associated with all the models are reported in Table 5. Model G1 (configural invariance) with no invariance constraints provided excellent fit to the data (CFI = 0.985, TLI = 0.981, RMSEA = 0.020). This result suggests that the structure was the same across gender groups. Then, Model G2 put equality constraints on factor loadings and thresholds. Differences between fit indices of Models G1 and G2 were negligeable (ΔCFI = −0.001, ΔTLI = +0.001, ΔRMSEA = 0.000), supporting strong invariance across gender groups. In Model G3, equality constraints were put on item uniquenesses. Differences between fit indices of Models G2 and G3 were negligeable (ΔCFI = +0.002, ΔTLI = +0.003, ΔRMSEA = −0.002), supporting strict invariance across gender groups. When equality constraints were placed on the latent variance-covariance matrix (Model G4), model fit did not decrease substantially between Models G3 and G4 (ΔCFI = +0.002, ΔTLI = +0.003, ΔRMSEA = −0.001), supporting latent variance-covariance invariance across gender groups. Finally, when latent means were constrained to be equal across gender groups (Model G5), differences between fit indices of Models G4 and G5 were again negligeable (ΔCFI = +0.001, ΔTLI = +0.001, ΔRMSEA = −0.001), supporting latent means invariance.

**Table 5:** Measurement invariance across gender for the bifactor-ESEM representation of early numeracy with one global factor and three specific facets (Model 8).

**Models**	**Chi-square**	**df**	**CFI**	**TLI**	**RMSEA [90% CI]**
**Gender invariance**					
G1. Configural	1413.160^**^	394	0.985	0.981	0.020 [0.014;0.025]
G2. Strong	1564.305^**^	251	0.984	0.982	0.020 [0.013;0.025]
G3. Strict	1585.352^**^	211	0.986	0.985	0.018 [0.011;0.023]
G4. Latent variance covariance	1572.469^*^	201	0.988	0.987	0.017 [0.009;0.022]
G5. Latent means	1568.819^*^	197	0.989	0.988	0.016 [0.008;0.022]

** *p ≤ 0.01*,

* *p ≤ 0.05; df, degrees of freedom; CFI, Comparative Fit Index; TLI, Tucker-Lewis Index; RMSEA, Root Mean Square Error of Approximation; CI, Confidence Interval*.

## Discussion

This study applied the integrative psychometric framework developed by Morin et al. (2016a) in order to investigate sources of construct-relevant multidimensionality of early numeracy and to test the superiority of the bifactor-ESEM of early numeracy. As stated by Gu et al. (2020), by overcoming the shortcomings of both the CFA and the ESEM models, the bifactor-ESEM is theoretically the most comprehensive and flexible model, able to describe more accurately the complex psychological characteristics.

In a first step, ICM-CFA and ESEM models with one, two or three sub-dimensions were compared. The ESEM solution provided a better fit to the data and lower factor correlations when compared to the ICM-CFA solution. In a second step, the ESEM model was compared to its bifactor counterpart (bifactor-ESEM). Among all models tested, the bifactor-ESEM solution with one general dimension and three specific facets (specific counting, specific relations, specific arithmetic) showed the best empirical fit with factors moderately well-defined, with less and lower significant cross loadings. A closer look to the loadings and cross-loadings on the specific counting and the specific relations factors lead to consider that four counting items (asking to count not starting from one) and four relations items (with perceptive bias) could be very specific and maybe represent independent specific counting and relations factors.

Data and analyses supported our hypothesis that early number skills could be underpinned by a latent common general factor; when the shared variance between the items is taken into account, specific numerical facets remain. The bifactor nature of early number skills had already been suggested by Ryoo et al. (2015) but using a bifactor-CFA model. However, the bifactor-CFA neglects the possibility that items may have cross-loadings on the non-target specific factors, which might result in inflated G-factor loadings (Murray and Johnson, 2013; Morin et al., 2016a). In contrast, the current study showed empirical support for the bifactor-ESEM solution as a comprehensive model of early numeracy. The explained common variance showed that the general factor explained 75% of the common variance extracted while 25% of the common variance was spread across the three specific factors. This suggests the existence of a general factor underlying all items of the test while taking into consideration the items' cross-loadings, which provides more accurate estimates of factor correlations and thereby better discriminant validity (Asparouhov and Muthén, 2009; Morin et al., 2016a). In practical terms, it means that factor scores (computed from the parameter estimates of the bifactor-ESEM solution) should be preferred to classical scale scores (computed by sum or average of items scores) which are unable to take cross-loadings into account and to adequately disaggregate the variance attributed to the general and the specific factors. Thus, the current study highlighted that early number skills—at least the ones that were measured here—might be considered as a multidimensional hierarchical construct that is underpinned by a single common factor and has valuable specific facets.

### Specific Facets

In the current study, when keeping under control the shared variance among items, several specific numerical facets emerged. In other words, the specific factors explain the shared variance among items that remains after controlling the effect of the general factor. This is of particular importance because the interpretation of specific factors in bifactor-ESEM differs from the typical interpretation of correlated factors in ICM-CFA, as they account for very different parts of the observed variance among items. With regards to this important difference, it is nevertheless interesting to note that our ICM-CFA, ESEM and bifactor-ESEM models are rather in line with the tripartite model of early numeracy supported by the NRC (Numbering, Relations and Arithmetic) than with the bipartite model supported by the NCTM (Numbers and Operations), since our data suggest three correlated factors (in ICM-CFA and ESEM) or one general dimension and three specific facets (in bifactor-ESEM) of early number skills. The counting factor (or specific counting facet) includes most of the verbal counting and object counting items, and is therefore quite similar to the Numbering dimension of the NRC, defined as the children's knowledge of the rules and processes of the counting sequence and the ability to obtain quantity in a flexible manner (Purpura and Lonigan, 2013). However, while the NRC Numbering dimension explicitly includes counting forward and backward from numbers other than one, our four items that required the children to count from numbers other than one seem to have a particular behavior. In other studies, in which items requiring counting forward from numbers other than one were included (Aunio and Niemivirta, 2010; Purpura and Lonigan, 2013), factor solutions with more than three dimensions were not explored. Thus, our results suggest than it could be interesting to distinguish more than three factors, considering that the ability to count from numbers other than one might constitute an ability *per se*. This factor might correspond to the breakable level of verbal counting mastery, as described by Fuson (1988). This level of verbal counting mastery is necessary to count from numbers other than one, while counting up to and enumerating collections can be done within an unbreakable list level of verbal string mastery. Therefore, such counting tasks might constitute a second specific counting dimension which could correspond to a higher level of counting skills than the first one. More studies are needed to investigate this hypothesis.

Our data support a specific Relations dimension, but again, several items appeared having a particular behavior. These items were involving a perceptual bias: the conservation of number task, and the three items in which children had to compare two collections with a perceptual trap. Perceptual bias occurs when the visuospatial appearance makes children think there are more elements in a first collection than in a second one, when in fact there are the same number in both collections or there are more elements in the second. This perceptual bias typically occurs when items of one of the collections are more dispersed. In order to succeed in this kind of tasks, children must inhibit the inappropriate “length-equals-number heuristic” (Bjorklund and Harnishfeger, 1990; Houdé et al., 2011), or more generally the visuospatial heuristic according to which “bigger is more.” It has been found that children are hardly capable of resisting these before the age of 7 years-old, leading to a failure in grasping the number principle *per se*. This can be explained by the fact that in many daily life situations, numerosity and occupied space are strongly linked, as more objects usually occupy more space. Houdé et al. (2011), by comparing the brain activation in children who were conservers (9–10 years old) and non-conservers (5–6 years old) in the Piagetian sense, found that while both groups of children processed the Piagetian task as a quantitative number task, activation in cerebral regions underpinning executive control, spatial working memory and episodic memory retrieval were necessary to resist the perceptual bias in order to succeed the task. Thus, it seems that the ability to refer to the cardinal property of numbers beyond the misleading perceptual characteristics of the items (Gelman and Gallistel, 1978) corresponds to a specific dimension of early number skills. The perceptual appearance being very influential at the age of our participants, these tasks could also assess an ability that relies on “a large-scale executive brain network” (Houdé et al., 2011; p. 344) rather than on typically numerical skill. Again, further studies are needed in this direction.

Finally, the last specific factor that emerged in the bifactor-ESEM solution is relatively well-defined by the different items planned to assess children's arithmetical skills. This one is quite similar to the NRC Arithmetic and to the NCTM Operation dimensions, defined as the understanding of the ways in which groups are composed and decomposed by differentiating sets and subsets (Purpura and Lonigan, 2013). Interestingly, in the studies analyzing the factorial structure of number skills that included arithmetic items, such a dimension was always extracted (Aunio et al., 2006; Aunio and Niemivirta, 2010; Purpura and Lonigan, 2013; Ryoo et al., 2015). Our study extends this finding through the bifactor-ESEM framework by showing that when keeping under control the common general factor that underlines early number skills, arithmetic problem responses are psychometrically distinct from other responses. However, the items loading on this specific dimension were more or less high. It should be specified that the items belonging to this specific facet were considerably different from each other. Indeed, the arithmetical tasks included in the test required the children to solve number combinations with concrete elements, to solve story problems and to decompose numbers. Thus, it could be that with more arithmetical subtests and items than in the current study, more specific arithmetical facets would appear beyond the control of the general factor.

### General Factor

The current results also indicate that a considerable part of variance was shared across all items of the test used, suggesting the existence of a general factor underlying early number skills. But what this latent common factor is still needs to be identified. This remains difficult since latent common factors can be domain-general abilities, domain-specific abilities, or even a mix of them. Since we did not measure general cognitive abilities in the current study, we can only formulate some hypotheses comparing our numerical data to the literature. In the case of early number skills, the domain-general factors that could be candidates for the latent general factor identified are the covariates that have been found to be associated to early number skills, such as general intelligence (Dickerson Mayes et al., 2009; Krajewski and Schneider, 2009; Green et al., 2018), working memory (Bull et al., 2008; Alloway and Alloway, 2009), linguistic skills (Kleemans et al., 2011; Raghubar and Barnes, 2017), executive functions (Espy et al., 2004; Kroesbergen et al., 2009), and visuo-spatial ability (Kyttälä et al., 2003). By looking at the items that loaded the more and the less on the general factor and by knowing from literature by which factors these numerical abilities are strongly underpinned, some suppositions can be made about what this general factor might most likely correspond to. The items that loaded the most on the general factor were verbal counting, counting small quantities, comparing/ordering few and small quantities, adding few concrete elements and solving simple problems (type change involving a small addition). On the opposite, the items that loaded the less on the general factor were adding higher quantities of concrete elements (for instance 7 and 5), complex arithmetic tasks such as number decomposition, difficult verbal problems (type change involving a subtraction or type combine), counting higher collections (12 or more) and conservation of numbers. Because complex arithmetic tasks and word problems in particular are known to be strongly underpinned by working memory (Geary et al., 2004; Träff, 2007; Zheng et al., 2011), language skills (Van Rinsveld et al., 2015) and general intelligence (Hornung et al., 2014), these factors do not appear to be the best candidates for the general factor highlighted here. The exclusion of language skills as a potential general factor is also supported by the fact that such skills were found not to predict non-linguistic arithmetic (LeFevre et al., 2010), items that highly loaded on the general factor in the current study. The observation that conservation of numbers did not strongly load on the general factor gives reason to think that executive functions are not either a good candidate for the general factor. Indeed, the ability to solve this task has been found to strongly rely on the executive function of inhibitory control (Houdé et al., 2011, see above). A general cognitive factor that might correspond to the current general factor would be visuo-spatial ability, since both non-linguistic arithmetic (LeFevre et al., 2010) and counting knowledge (Kyttälä et al., 2003)—which strongly loaded on it here—were previously found to be related to visuospatial skills.

The latent common factor found in the current study could also be a more domain-specific ability. Interestingly, Mou et al. (2021), who showed the superiority of a bifactor-CFA by measuring simultaneously numerical and more general abilities, highlighted that only one third of their general latent factor variance was explained by the combination of general non-numerical dimensions, such as cognitive and linguistic abilities and age. This suggested that the general factor they found was mainly numerical. From the literature, a domain-specific factor that could be a candidate for the latent general factor found here is number sense. It refers to elementary intuitions about quantity, such as the rapid and accurate perception of small numerosities, the ability to compare roughly larger numerical magnitudes, to count, and to comprehend simple arithmetic operations, and is considered as an essential tool to develop higher order mathematical skills (Case et al., 1992; Geary, 1995; Dehaene, 1997, 2001; Berch, 2005; Gersten et al., 2005; Jordan et al., 2010; Chu et al., 2015; Sowinski et al., 2015). In the current study, the fact that the items loading the more on the general factor were very fundamental skills gives credit to the possibility that number sense corresponds to this general factor.

The last hypothesis is that the general factor would only be a psychometric artifact due to the positive manifold of correlations observed between cognitive test tasks (van der Maas et al., 2006, 2011; Borsboom, 2017; Fried et al., 2017). According to van Bork et al. (2017), “the existence of a general factor is not tested against the data because any dataset that features a positive manifold will necessarily support a general factor model, whether or not a general factor underlies the data” (p. 764). This is the main argument of the precited authors who developed the relatively recent Network Analysis approach, somehow rejecting the latent variable approach (for a critique of this position, see Guyon et al., 2017). The proponents of Network Analysis offer an alternative explanation for the positive intercorrelations between tasks scores, based on the biological concept of mutualism (van der Maas et al., 2006). This concept postulates that the positive manifold emerges purely by mutually beneficial relationships between cognitive processes and its developmental dynamic. In concrete words, cognitive abilities (the nodes) are conceptualized as being more or less directly related to each other (the edges) without postulating any latent factor. Recently, Kan et al. (2019) and Kan et al. (2020) have shown the superior fit of a network model of intelligence compared to a higher-order *g* model and a bifactor-CFA model. As Network Analysis is not implemented in MPlus and currently not available with binary variables within the reference R package *Psychonetrics* (Epskamp, 2020a,b), we did not have the opportunity to compare the ICM-CFA and ESEM models with a Network Analysis model of early numeracy. Anyway, this would not have provided a definitive answer to the question of which statistical model best represents reality, as the aim in the social sciences is to obtain the best theoretically and practically useful representation of what is under investigation. Importantly, authors of the Network Analysis approach have never compared network models with ESEM and bifactor-ESEM models. Our results provide evidence that both sources of construct-relevant psychometric multidimensionality were present in the early numeracy test used, supporting the use of ESEM and suggesting the appropriateness of the bifactor-ESEM, with regards to traditional ICM-CFA models. ICM-CFA and ESEM solutions differed indeed in their factor correlations with much lower factor correlations for ESEM than for ICM-CFA. Including cross-loadings in ESEM decreases the amount of shared variance among items (i.e., the positive manifold), suggesting that ESEM resulted in a better differentiation between early numeracy factors than ICM-CFA. Moreover, the existence of significant and substantial non-target cross-loadings in ESEM supported the need for a bifactor-ESEM analysis. Examination of the bifactor-ESEM solution revealed that many fewer items were presenting meaningful and substantial non-target cross-loadings compared to ESEM solutions. On this basis, and because factor loadings on the general factor are large, and correlations between the specific factors scores are non-significant within the bifactor-ESEM solution (see Morin et al., 2020), we would support that the general factor underlined in this study is not a psychometric artifact, and not better or worse than alternative equivalents models (MacCallum et al., 1993; Williams, 2012).

## Limitations, Conclusion, and Further Prospects

There are several limitations to this study. First, the sample of children was a convenience sample. Even if we tried to enroll schools with socio-economically diverse background, the sample is not representative, and results cannot be generalized. Secondly, within-participants variations were not examined across time. It would be interesting to know if the bifactor-ESEM structure of early number skills changes over time or not. Third, pupils in our study were assessed through a French-speaking test. Further studies should confirm if the bifactor-ESEM representation of early numeracy is still adequate when English-speaking tests are used.

The present study highlighted the relevance of the bifactor-ESEM framework when it comes to better identifying the dimensionality of early number skills. Indeed, it was found from the current data that when keeping under control the high intercorrelations within that domain, three specific numerical dimensions remained. These specific facets of early numeracy, which are different in nature compared to correlated factors in ICM-CFA or ESEM, have a substantive value. However, the specific facets highlighted here should not obscure the fact that early numeracy appears to be principally a unidimensional construct.

Determining the structure of early numeracy skills is crucial to assessing them properly, as well as teaching them efficiently, developing appropriate early intervention strategies for at-risk children, and studying their relations with other variables. For instance, if the structure of early numeracy that stood out in the current experiment is confirmed by further studies, this suggests that when seeking to assess the number skills of young children, subtests measuring both the general latent factor and the three highlighted specific dimensions should minimally be taken into consideration. The refined investigation of early numeracy structure conducted here could also lead to more targeted teaching activities for all pupils and to reinforcement activities for pupils identified as at risk of school failure, as it has been done in the domain of early literacy (Juel and Minden-Cupp, 2000). Concretely, teaching materials could be developed to enhance pupils' skills on the general factor and the specific dimensions. Also, as pointed by Brunner (2008), the relationships between math abilities and other variables strongly depends on the structural conception of math skills. For instance, the relationships between transversal variables such as socioeconomic status or general intelligence could be different with the general or the specific math dimensions highlighted in the current study. This could lead to identify some math abilities that are more dependent on children's cognitive characteristics or socioeconomic heritage, and others that could be more dependent, for example, on the instruction received.

In order to further investigate the dimensionality of early numeracy and in particular to better identify what the current general factor could be, a bifactor-ESEM analysis could be conducted on additional early number skills tasks as well as on tasks measuring the more general candidates. For example, number sense is typically assessed by the speed with which individuals make magnitude comparisons, or by their ability to place numbers on a number line (Berch, 2005; Siegler and Ramani, 2009), skills that were not assessed here. Including such tasks in a further study as well as general abilities such as non-verbal intelligence, working memory, etc., and observing how the numerical items would behave on the general factor, would allow to identify more reliably what this general factor could correspond to. Further studies could also address the predictive validity of the bifactor-ESEM solution of early numeracy. Demonstrating it would strengthen the relevance and the added value of such a model.

In the future, it also seems to us that to diminish sources of confusion in the understanding of the early number skills structure, harmonization efforts should be made regarding the selected tasks and their headings. For example, while several authors included arithmetic tasks in their studies (Aunio and Niemivirta, 2010; Purpura and Lonigan, 2013; Ryoo et al., 2015), other researchers did not (Cirino, 2011; Hirsch et al., 2018). This can, of course, have an incidence on the number of factors that stand out. The number of tasks among each type of skills is also very different from study to study, which is known to affect the factor structure (Purpura and Lonigan, 2013). For example, Lee et al. (2012) administered a 1 min number facts task as a measure of arithmetic ability, while Purpura and Lonigan's (2013) included eight different arithmetic tasks comprising 38 items in total. An additional source of confusion in the factors identified might come from the use—or not—of Arabic numerals. Indeed, some authors used several tasks involving digits (i.e., Cirino, 2011; Purpura and Lonigan, 2013; Ryoo et al., 2015) while others did not (Aunio and Niemivirta, 2010) or only a few (Hirsch et al., 2018). Actually, the difference between symbolic and non-symbolic skills has been found to be a major distinction (Cirino, 2011). Since tasks involving Arabic numerals are generally considered as falling within the relations dimension (National Research Council, 2009; Cirino, 2011; Purpura and Lonigan, 2013), the interlacing between this dimension and Arabic numerals might, maybe improperly, strengthen the incidence of this dimension. Finally, the headings of the subtests are also sometimes confusing. For example, while the counting tasks used by the majority of the authors consist of counting forward or backward, or in enumerating collections and are considered as belonging to the Number dimension, the Hirsch et al. (2018) “counting task” requires the children to draw a line below the *n*th bell among a line of bells, and this appeared as an eponymous factor *per se*. This task is therefore very similar to the subtest called “ordinality” in Purpura and Lonigan's (2013) study (identify the *n*th picture in a line) that was part of their relations dimension.

In sum, a promising avenue to further study the early number skills dimensionality is to apply the bifactor-ESEM framework on data including candidates for the general factor, as well as by taking into account as comprehensively as possible specific facets that might stand out. Precisely understanding what the important general factor underlying early number skills is, and the exact specific dimensions that remain after having kept under control the high intercorrelations within that domain, would constitute significant progress in the study of early numeracy. This analysis could be interestingly contrasted with a network analysis in order to discard the psychometric artifact hypothesis that several authors are supporting concerning general latent factor models.

## Data Availability Statement

The raw data supporting the conclusions of this article will be made available by the authors, without undue reservation.

## Ethics Statement

The study was reviewed and approved by the Ethics Review Panel of the University of Luxembourg in charge of study coordination. The legal guardian of the pupils provided written informed consent to participate in this study.

## Author Contributions

CD and AFC wrote the manuscript. All authors contributed to the conception of the study, the manuscript revision, and approved the submitted version.

## Conflict of Interest

The authors declare that the research was conducted in the absence of any commercial or financial relationships that could be construed as a potential conflict of interest.

## Footnotes

^1^In a second-order (or higher-order) model, paths are specified from a second-order factor to the first-order factors, which in turns have paths leading to observed indicators. Second-order model relies on stringent proportionality constraints which are nevertheless unlikely to hold in practice (for details, see Canivez, 2016; Morin et al., 2016a).

^2^An Item Response Theory (IRT) model including difficulty and discrimination parameters (Birnbaum, 1968) was used to estimate pupil competence and item parameters of the test (for details, see Masked Reference). As an alternative to the classical theory (or true score theory) where a total test score is calculated simply by adding correct answers (but with measurement error), IRT modeling estimates the probability that a student will give a correct answer to a specific item, depending both on the student ability and the specific items characteristics, leading to a weighted score (for more details about IRT, see van der Linden, 2017). The analyses were conducted using Conquest 2.0 software, which generated a Warm's mean likelihood estimation (WLE) for each pupil. On 41 items, one (7_2) was *a posteriori* discarded from the analyses because of bad item fit statistics.
