# Supplementary material for: Investigating the Dimensionality of Early Numeracy Using the Bifactor Exploratory Structural Equation Modeling Framework
Source: Front Psychol. 2021 Jun 22;12:680124. doi: 10.3389/fpsyg.2021.680124 (PMC8258407; doi:10.3389/fpsyg.2021.680124)
Supplement: Supplementary file 1 [file Table_1.docx]

**Appendix 1: Overview of previous dimensionality studies of early numeracy**

**Table A1**

*Factorial structures of early numeracy in previous dimensionality studies*

| Study | Sample size and age | Test used | Model(s) tested, and model finally recommended by authors (in bold) | Main fit indexes reported | | | Sub-dimensions of the best fitting model | Measurement invariance | Comments or methodological concerns |
| --- | --- | --- | --- | --- | --- | --- | --- | --- | --- |
|  |  |  |  | RMSEA | CFI | PPP |  |  |  |
| Hellstrand, Korhonen, Räsänen, Linnanmäki, and Aunio (2020) | Kindergarten – 361 – Mean age (73.97 months) and SD (3.65) | EN-test (Koponen et al., 2011a, 2011b, 2011c) | **4-factor (first-order)** | .029 | .935 | - | 1. Symbolic and non-symbolic number knowledge  2.Understanding mathematical relations  3. Counting skills  4. Basic skills in arithmetic | Invariance across gender, age, and language versions. | First-order factors highly correlated with each other. |
|  |  |  | 3-factor (first-order) | .034 | .910 | - |  |  |  |
|  |  |  | 1-factor | .039 | .881 | - |  |  |  |
|  | Grade 1 – 321 – Mean age (86.87 months) and SD (3.94) |  | **4-factor (first-order)** | .034 | .885 | - | 1. Symbolic and non-symbolic number knowledge  2.Understanding mathematical relations  3. Counting skills  4. Basic skills in arithmetic |  |  |
|  |  |  | 3-factor (first-order) | .037 | .865 | - |  |  |  |
|  |  |  | 1-factor | .046 | .790 | - |  |  |  |
|  | Grade 2 – 457 – Mean age (98.38 months) and SD (3.65) |  | **4-factor (first-order)** | .028 | .967 | - | 1. Symbolic and non-symbolic number knowledge  2.Understanding mathematical relations  3. Counting skills  4. Basic skills in arithmetic |  |  |
|  |  |  | 3-factor (first-order) | .030 | .964 | - |  |  |  |
|  |  |  | 1-factor | .046 | .934 | - |  |  |  |
| Lopez-Pedersen, Mononen, Korhonen, Aunio & Melby-Lervåg (2020) | 366 – Mean age (6.36 years) and SD (not reported) | Early Numeracy Screener | **3-factor (first-order)** | .06 | .911 | - | 1. Counting skills  2. Numerical relations  3. Arithmetic | Invariance across gender and age. | High correlations between first-order factors. |
|  |  |  | 1-factor | .01 | .938 | - |  |  |  |
| Braeuning, Ribner, Moeller, and Blair (2020) | 846 – Mean age (60.16 months) and SD (3.29) | Early Childhood Longitudinal Study-Kindergarten (ECLS-K) math assessment (Rock, Pollack, & Hausken, 2002) | 6-factor (first-order) | .029 | .946 | - | 1. Patterning and geometry  2. Number sense  3. Arithmetic  4. Data analysis and statistics. | Configural but not metric invariance across time (pre-K to K) | Inclusion of geometry and graphs analysis items.  High correlations between first-order factors. |
|  |  |  | **4-factor (first-order)** | .033 | .962 | - |  |  |  |
|  |  |  | 1-factor | .038 | .904 | - |  |  |  |
| Milburn, Lonigan, DeFlorio, and Klein (2018) | 1630 – Mean age (4.46 years) and SD (.37) | Child Math Assessment (Starkey, Klein & Wakeley, 2004)  35 items | **3-factor (first-order)** | .04 | .97 | - | 1. Numbering  2. Relations  3. Operations | Not tested. | Correlations between first-order factors not reported. |
|  |  |  | 2-factor (first-order) | .06 | .94 | - |  |  |  |
|  |  |  | 2-factor (first-order) | .06 | .95 | - |  |  |  |
|  |  |  | 2-factor (first-order) | .07 | .93 | - |  |  |  |
|  |  |  | 1-factor | .08 | .90 | - |  |  |  |
| Hirsch, Lambert, Coppens, and Moeller (2018) | 1727 – 5 to 6 years old | Selection of 32 items from the Ordenen-Oudste kleuters (CITO, 1997) | **5-factor (first-order)** | .014 | .97 | - | 1. Patterning  2. Seriation  3. Non-symbolic comparison  4. Counting  5. Symbolic number knowledge | Not tested. | Use of a covariate (general cognitive ability) in the CFA. |
|  |  |  | 4-factor (first-order) | .013 | .97 | - |  |  |  |
|  |  |  | 2-factor (first-order) | .017 | .95 | - |  |  |  |
|  |  |  | 1-factor | .020 | .93 | - |  |  |  |
| Cirino (2011) | 285 - Mean age (6.13 years) and SD (.3) | Original items and selection of items from AIMSweb (Clarke & Shinn, 2002)  12 indicators | **5-factor (first-order)** | .075 | .944 | - | 1. Non-symbolic comparison  2. Symbolic comparison  3. Symbolic labeling  4. Rote counting  5. Counting knowledge | Not tested. | RMSEA above .05 and CFI values below .95. |
|  |  |  | 4-factor (first-order) | .080 | .933 | - |  |  |  |
|  |  |  | 3-factor (first-order) | .098 | .891 | - |  |  |  |
|  |  |  | 2-factor (first-order) | .108 | .863 | - |  |  |  |
|  |  |  | 1-factor | .123 | .819 | - |  |  |  |
| Purpura, and Lonigan (2013) | 393 -  37 to 72 months | PENS (Purpura, 2010)  25 tasks, each with 3 to 9 items | **3-factor (first-order)** | .05 | .95 | - | 1. Numbering  2. Relations  3. Operations | Invariance across age group, but exact results of the multi-group analysis were not reported. | Separate testing sessions (3 subtests).  Correlated residuals.  First-order factors highly correlated with each other. |
|  |  |  | 2-factor (first-order) | .06 | .95 | - |  |  |  |
|  |  |  | 2-factor (first-order) | .06 | .94 | - |  |  |  |
|  |  |  | 2-factor (first-order) | .06 | .94 | - |  |  |  |
|  |  |  | 1-factor | .06 | .94 | - |  |  |  |
| Aunio, Ee, Lim, Hautamäki, and Van Luit (2004) | 630 (in 3 countries) – Mean age (73 months) and SD (1.26) | ENT (Van Luit et al., 1994)  40 items | **2-factor (first-order)** | .046 | .97 | - | 1. Counting skills  2. Relational skills | Not tested. | First-order factors highly correlated with each other. |
|  |  |  | 1-factor | .052 | .96 | - |  |  |  |
| Aunio, and Niemivirta (2010) | 212 - Mean age (72.2 months) and SD (3.49) | ENT (Van Luit et al., 2006)  40 items | **2-factor (first-order)** | .04 | .95 | - | 1. Counting skills  2. Relational skills | Not tested. | First-order factors highly correlated with each other. |
| Aunio, Korhonen, Ragpot, Törmänen, Mononen, and Henning (2019) | 443 – Mean age (81.6 months,) and SD (5.40) | ThinkMath (Aunio & Mononen, 2012)  43 items | **3-factor (first-order)** | .059 | .912 | - | 1. Counting tasks  2. Numerical relational tasks  3. Arithmetical word problem tasks | Not tested. | Test unpublished.  RMSEA above .05 and CFI values below .95. |
|  |  |  | 2-factor (first-order) | .059 | .905 | - |  |  |  |
|  |  |  | 1-factor | .061 | .901 | - |  |  |  |
| Mou, Zhang, Piazza, and Hyde (2021) | 204 – Mean age (46.5 months) and SD (61 days) | Modified computer versions of the Give-N task (Wynn, 1992) and the How-Many? Task (Le Corre et al. 2006; Wynn, 1992). | **3-factor (bifactor)** | .083 | .940 |  | General factor  1. Give-N  2. How-Many? | Not tested. | RMSEA above .05. |
|  |  |  | 2-factor (first-order) | .111 | .874 |  |  |  |  |
|  |  |  | 1-factor | .135 | .811 |  |  |  |  |
| Ryoo, Molfese, Brown, Karp, Welch, and Bovaird (2015) | Fall 07:  389 – 47 to 59 months | Selections of items from the TEMA-3 (Ginsburg & Baroody, 2003)  Fall 07: 32 items  Spring 08: 41 items  Spring 09: 56 items  Spring 10: 62 items | 1-factor | .034 | - | - | General factor  1. Counting objects  2. Verbal counting  3. Numerical comparison  4. Set construction  5. Numeral literacy  6. Calculation | Not tested. | Use of Bayesian CFA because of estimation problems in CFA models using WLSMV estimator. |
|  |  |  | 7-factor (first-order/Bayesian CFA) | - | - | .350 |  |  |  |
|  |  |  | 7-factor (second-order) | - | - | .307 |  |  |  |
|  |  |  | **7-factor (bifactor)** | - | - | .404 |  |  |  |
|  |  |  | 8-factor (first-order/Bayesian CFA) | - | - | .379 |  |  |  |
|  |  |  | 8-factor (second-order) | - | - | .250 |  |  |  |
|  |  |  | 8-factor (bifactor) | - | - | .339 |  |  |  |
|  | Spring 08:  360 – 51 to 67 months |  | 1-factor | .064 | - | - | General factor  1. Counting objects  2. Verbal counting  3. Numerical comparison  4. Set construction  5. Numeral literacy  6. Calculation |  |  |
|  |  |  | 7-factor (first-order/Bayesian CFA) | - | - | .278 |  |  |  |
|  |  |  | 7-factor (second-order) | - | - | .220 |  |  |  |
|  |  |  | **7-factor (bifactor)** | - | - | .284 |  |  |  |
|  |  |  | 8-factor (first-order/Bayesian CFA) | - | - | .242 |  |  |  |
|  |  |  | 8-factor (second-order) | - | - | .189 |  |  |  |
|  |  |  | 8-factor (bifactor) | - | - | .058 |  |  |  |
|  | Spring 09:  306 – 63 to 76 months |  | 1-factor | .053 | - | - | 1. Counting objects  2. Verbal counting  3. Numerical comparison  4. Set construction  5. Numeral literacy  6. Calculation  7. Number facts |  |  |
|  |  |  | **7-factor (first-order/Bayesian CFA)** | - | - | .399 |  |  |  |
|  |  |  | 7-factor (second-order) | - | - | .348 |  |  |  |
|  |  |  | 7-factor (bifactor) | - | - | .384 |  |  |  |
|  |  |  | 8-factor (first-order/Bayesian CFA) | - | - | .357 |  |  |  |
|  |  |  | 8-factor (second-order) | - | - | .283 |  |  |  |
|  |  |  | 8-factor (bifactor) | - | - | .350 |  |  |  |
|  | Spring 10:  294 – 78-91 |  | 1-factor | .037 | - | - | General factor  1. Counting objects  2. Verbal counting  3. Numerical comparison  4. Set construction  5. Numeral literacy  6. Calculation  7. Number facts |  |  |
|  |  |  | 7-factor (first-order/Bayesian CFA) | - | - | .272 |  |  |  |
|  |  |  | 7-factor (second-order) | - | - | .147 |  |  |  |
|  |  |  | 7-factor (bifactor) | - | - | .211 |  |  |  |
|  |  |  | **8-factor (first-order/Bayesian CFA)** | - | - | .311 |  |  |  |
|  |  |  | 8-factor (second-order) | - | - | .116 |  |  |  |
|  |  |  | 8-factor (bifactor) | - | - | .214 |  |  |  |

As reported in Table A1, comparing four first-order factor models to the one-factor model, Cirino (2011) found superior empirical evidence for a 5-factor ICM-CFA structure with non-symbolic comparison, symbolic comparison, symbolic labelling, rote counting, and counting knowledge as first-order factors. In five studies (Aunio, Ee, Lim, Hautamäki, & Van Luit, 2004; Aunio & Niemivirta, 2010; Aunio, Korhonen, Ragpot, Törmänen, Mononen, & Henning, 2019; Hellstrand, Korhonen, Räsänen, Linnanmäki, & Aunio, 2020; Lopez-Pedersen, Mononen, Korhonen, Aunio, and Melby-Lervåg, 2020), Aunio and colleagues have compared ICM-CFA models to the one-factor model. In the first study, they concluded that the one-factor model and the 2-factor ICM-CFA model (with number relations skills and counting skills as correlated first-order factors) were “two almost equally good ways of assessing number sense” (p. 211). In the second study, they only tested the 2-factor ICM-CFA model and concluded that this factorial solution fitted the data well. In the third study, they used another test (ThinkMath, unpublished test) and highlighted the superiority of a 3-factor ICM-CFA model with number relations skills, counting skills and arithmetical word problems skills as correlated first-order factors. In the fourth study using the Early Numeracy tests (Koponen et al., 2011a, 2011b, 2011c), they showed the superiority of a 4-factor ICM-CFA model for Kindergarten, grade 1 and grade 2 with symbolic and non-symbolic number knowledge, understanding mathematical relations, counting skills, and basic skills in arithmetic. They nevertheless highlighted in the study limits that these four factors were highly correlated and that some tasks assessing specific dimensions were partially similar and conceptually overlapping. In the fifth study, Lopez-Pedersen, Mononen, Korhonen, Aunio, and Melby-Lervåg (2020) validated their Early Numeracy Screener for first graders. They showed that the best fitting model was the 3-factor ICM-CFA model with counting skills, numerical relations and arithmetic. Purpura and Lonigan (2013) also concluded on the superiority of a 3-factor ICM-CFA model with highly correlated first-order factors (numbering, relations, operations). This 3-factor ICM-CFA model has been replicated by Milburn, Lonigan, DeFlorio and Klein (2019), while showing, however, that these three first-order factors could be underpinned by a second-order factor of numeracy when also considering items assessing geometry, patterning and measurement. For their part, Hirsch, Lambert, Coppens and Moeller (2018) compared three ICM-CFA models to the one-factor model. By including a measure of general cognitive ability as a covariate in the CFA, they found that the 5-factor ICM-CFA structure with patterning, seriation, non-symbolic comparison, counting, and symbolic number knowledge as first-order factors fitted the data best. Using the ECLS-K (Rock and Pollack, 2002) in Pre-K, Braeuning, Ribner, Moeller, and Blair (2020) identified a 4-factor ICM-CFA model as the best fitting model with patterning/geometry, number sense, arithmetic, and data analysis/statistics. Except for data analysis/statistics dimension, correlations between the other dimensions were very high.

**References**

Aunio, P. & Mononen, R. (2012). *ThinkMath-scale, Kindergarten*. Unpublished.

Aunio, P., & Niemivirta, M. (2010). Predicting children’s mathematical performance in grade one by early numeracy. *Learning and Individual Differences*, *20*, 427-435. <https://doi.org/10.1016/j.lindif.2010.06.003>

Aunio, P., Ee, J., Lim, S. E. A., Hautamäki, J., & Van Luit, J. (2004). Young children’s number sense in Finland, Hong Kong, and Singapore. *International Journal of Early Years Education*, *12*, 195-216. <https://doi.org/10.1080/0966976042000268681>

Aunio, P., Korhonen, J., Ragpot, L., Törmänen, M., Mononen, R., & Henning, E. (2019). Multi-factorial approach to early numeracy – The effects of cognitive skills, language factors and kindergarten attendance on early numeracy performance of South African first graders. *International Journal of Educational Research*, *97*, 65-76. <https://doi.org/10.1016/j.ijer.2019.06.011>

Braeuning D, Ribner A, Moeller K and Blair C (2020) The Multifactorial Nature of Early Numeracy and Its Stability. *Front. Psychol.* 11:518981. doi: 10.3389/fpsyg.2020.518981

Cirino, P. T. (2011). The interrelationships of mathematical precursors in kindergarten. *Journal of Experimental Child Psychology*, *4,* 713-733. <https://doi.org/10.1016/j.jecp.2010.11.004>

Cito (1997). *Leerlingvolgsysteem: Ordenen – Platenoek Oudste Kleuters* (Cito Ed.). Arnhem, Netherlands: Cito.

Clarke, B., & Shinn, M. R. (2002). *Test of Early Numeracy (TEN): Administration and scoring of AIMSweb Early Numeracy measures for use with AIMSweb*. Eden Prairie, MN: Edformation.

Ginsburg, H. P. & Baroody, A. J. (2003). *Test of Early Mathematics Ability, Third Edition* (TEMA-3). Austin, TX: Pro-Ed.

Hellstrand, H., Korhonen, J., Räsänen, P., Linnanmäki, K., & Aunio, P. (2020). Reliability and validity evidence of the early numeracy test for identifying children at risk for mathematical learning difficulties. *International Journal of Educational Research*, 102, https://doi.org/10.1016/j.ijer.2020.101580.

Hirsch, S., Lambert, K., Coppens, K., & Moeller, K. (2018). Basic numerical competences in large-scale assessment data: Structure and long-term relevance. *Journal of Experimental Child Psychology*, *167*. 32-48. <https://doi.org/10.1016/j.jecp.2017.09.015>

Koponen, T., Salminen, J., Aunio, P., Polet, J., & Hellstrand, H. (2011a). LukiMat - Bedömning av lärandet: Identifiering av stödbehov i matematik i förskola. Handbok [LukiMat – Assessment for Learning: Identifying Children in Need of Support in Mathematics in Kindergarten. Handbook]. <http://www.lukimat.fi/lukimat-bedomning-av-larandet/material/identifiering-av-stodbehov/forskola/f-mat-handbok>.

Koponen, T., Salminen, J., Aunio, P., Polet, J., & Hellstrand, H. (2011b). LukiMat - Bedömning av lärandet: Identifiering av stödbehov i matematik i årskurs 1. Handbok [LukiMat – Assessment for Learning: Identifying Children in Need of Support in Mathematics in First Grade. Handbook]. <http://www.lukimat.fi/lukimat-bedomning-av-larandet/material/identifiering-av-stodbehov/ak-1/1-mat-handbok>.

Koponen, T., Salminen, J., Aunio, P., Polet, J., & Hellstrand, H. (2011c). LukiMat - Bedömning av lärandet: Identifiering av stödbehov i matematik i årskurs 2. Handbok [LukiMat – Assessment for Learning: Identifying Children in Need of Support in Mathematics in Second Grade. Handbook]. http://www.lukimat.fi/lukimat-bedomning-av-larandet/material/identifiering-av-stodbehov/ak-2/2-mat-handbok.

Le Corre, M., Van de Walle, G., Brannon, E. M., & Carey, S. (2006). Re-visiting the competence/performance debate in the acquisition of the counting principles. *Cognitive Psychology*, 52, 130–169. http://dx.doi.org/10.1016/j.cogpsych.2005. 07.002

Lopez-Pedersen, A., Mononen, R., Korhonen, J., Aunio, P. & Melby-Lervåg, M. (2020) Validation of an Early Numeracy Screener for First Graders, *Scandinavian Journal of Educational Research*, https://doi.org/[10.1080/00313831.2019.1705901](https://doi.org/10.1080/00313831.2019.1705901)

Milburn, T. F., Lonigan, C. J., DeFlorio, L., & Klein, A. (2019). Dimensionality of preschoolers’ informal mathematical abilities. *Early Childhood Research Quarterly*, *47*, 487-495. <https://doi.org/10.1016/j.ecresq.2018.07.006>

Mou, Y., Zhang, B., Piazza, M., & Hyde, D. C. (2021). Comparing set-to-number and number-to-set measures of cardinal number knowledge in preschool children using latent variable modeling. *Early Childhood Research Quarterly, 45*, 125-135. <https://doi.org/10.1016/j.ecresq.2020.05.016>

Purpura, D. J. (2010). *Informal number-related mathematics skills: An examination of the structure of and relations between these skills in preschool* (Doctoral dissertation).

Purpura, D. J., & Lonigan, C. J. (2013). Informal numeracy skills: The structure and relations among numbering, relations, and arithmetic operations in preschool. *American Educational Research Journal*, *50*, 178-209. [https://doi.org/10.3102/0002831212465332](https://doi.org/10.3102%2F0002831212465332)

Rock, D. A., Pollack, J. M., and Hausken, E. G. (2002). *Early Childhood Longitudinal Study-Kindergarten Class of 1998-99 (ECLS-K): Psychometric Report for Kindergarten Through First Grade (NCES 2002-05)*, Washington, DC: National Center for Education Statistics, U.S. Department of Education.

Ryoo, J. H., Molfese, V. J., Brown, E. T., Karp, K. S., Welch, G. W., & Bovaird, J. A. (2015). Examining factor structures on the Test of Early Mathematics Ability **–** 3: A longitudinal approach. *Learning and Individual Differences*, *41*, 21-29. [https://doi.org/10.1016/j.lindif.2015.06.003](https://doi-org.proxy.bnl.lu/10.1016/j.lindif.2015.06.003)

Starkey, P., Klein, A., & Wakeley, A. (2004). Enhancing young children’s mathematical knowledge through a pre-kindergarten mathematics intervention. *Early Childhood Research Quarterly*, 19, 99-120. doi:10.1016/j.ecresq.2004.01.002

Van Luit, J. E. H., Van de Rijt, B. A. M., & Aunio, P. (2006). *Early Numeracy Test, Finnish Edition* [Lukukäsitetesti]. Helsinki, Finland: Psykologien kustannus.

Van Luit, J. E. H., Van de Rijt, B. A. M. & Pennings, A. H. (1994) *Utrechtse Gatalbegrip Toets*, UGT [Utrecht Test of Number Sense] (Doetinchem, Graviant).

Wynn, K. (1992). Children’s acquisition of the number words and the counting system. *Cognitive Psychology*, 24, 220–251. http://dx.doi.org/10.1016/0010- 0285(92)90008-P
